# Spousal bereavement and its effects on later life physical and cognitive capability: the Tromsø study

**DOI:** 10.1007/s11357-024-01150-y

**Published:** 2024-04-09

**Authors:** Bjørn Heine Strand, Asta K. Håberg, Harpa Sif Eyjólfsdóttir, Almar Kok, Vegard Skirbekk, Oliver Huxhold, Gøril Kvamme Løset, Carin Lennartsson, Henrik Schirmer, Katharina Herlofson, Marijke Veenstra

**Affiliations:** 1 Department for Physical Health and Aging, Norwegian Institute of Public Health, Oslo, Norway; 2 Norwegian National Centre for Ageing and Health, Vestfold Hospital Trust, Tønsberg, Norway; 3 Department of Geriatric Medicine, Oslo University Hospital, Oslo, Norway; 4 Department of Neuromedicine and Movement Science, Faculty of Medicine and Health Sciences, NTNU, Trondheim, Norway; 5 Aging Research Center (ARC), Karolinska Institutet, Stockholm, Sweden; 6 Centre of Public Health Sciences, Faculty of Medicine, University of Iceland, Reykjavik, Iceland; 7 Department of Epidemiology & Data Science, Amsterdam UMC, Vrije Universiteit Amsterdam, Amsterdam, The Netherlands; 8 Amsterdam Public Health, Aging & Later Life Programme, Amsterdam, The Netherlands; 9 DZA, Berlin, Germany; 10 NOVA – Norwegian Social Research, Oslo Metropolitan University, Oslo, Norway; 11 Swedish Institute for Social Research (SOFI), Stockholm University, Stockholm, Sweden; 12 Department of Clinical Medicine, UiT The Arctic University of Norway, Tromsø, Norway; 13 Department of Cardiology, Akershus, University Hospital, Lørenskog, Norway; 14 Institute of Clinical Medicine, University of Oslo, Oslo, Norway; 15 HØKH Akershus University Hospital, Lørenskog, Norway; 16 Stockholm University, Stockholm, Sweden

**Keywords:** Grip strength, Processing speed, Memory, Intrinsic capacity, Propensity score matching

## Abstract

Spousal bereavement is associated with health declines and increased mortality risk, but its specific impact on physical and cognitive capabilities is less studied. A historical cohort study design was applied including married Tromsø study participants (*N*=5739) aged 50–70 years with baseline self-reported overall health and health-related factors and measured capability (grip strength, finger tapping, digit symbol coding, and short-term recall) at follow-up. Participants had data from Tromsø4 (1994–1995) and Tromsø5 (2001), or Tromsø6 (2007–2008) and Tromsø7 (2015–2016). Propensity score matching, adjusted for baseline confounders (and baseline capability in a subset), was used to investigate whether spousal bereavement was associated with poorer subsequent capability. Spousal bereavement occurred for 6.2% on average 3.7 years (SD 2.0) before the capability assessment. There were no significant bereavement effects on subsequent grip strength, immediate recall, or finger-tapping speed. Without adjustment for baseline digit symbol coding test performance, there was a negative significant effect on the digit symbol coding test (ATT −1.33; 95% confidence interval −2.57, −0.10), but when baseline digit symbol coding test performance was taken into account in a smaller subsample, using the same set of matching confounders, there was no longer any association (in the subsample ATT changed from −1.29 (95% CI −3.38, 0.80) to −0.04 (95% CI −1.83, 1.75). The results in our study suggest that spousal bereavement does not have long-term effects on the intrinsic capacity components physical or cognition capability to a notable degree.

**Supplementary Information:**

The online version contains supplementary material available at 10.1007/s11357-024-01150-y.

## Introduction

The death of a spouse or partner is a life-changing, stressful event linked to increased mortality and morbidity of the surviving partner via several biological pathways [1–3]. Since spousal bereavement occurs most frequently among older adults [4], it coincides with the aging process and can influence a person’s intrinsic capacity [5] thereby affecting the trajectory of healthy aging. The concept intrinsic capacity, as introduced by The World Health Organization (WHO) in 2015 [6], is a composite of all the individual’s physical and mental capacities contributing to healthy ageing. WHO defines five domains of intrinsic capacity: cognition, sensory capacity (vision and hearing), locomotor capacity, vitality, and psychological capacity. The WHO has clarified the concepts and proposed operationalizations of the key intrinsic capacity domains [7], but different studies use a variety of measures. The vitality domain has been adopted as an umbrella term for physiological reserve resilience and biological age, and grip strength has recently been proposed as one of the key biomarkers to measure vitality [8].

The impact of physical capability on longevity is well established [9–12]. However, less is known about how major life transitions, such as bereavement, affect different measures of intrinsic capacity. In previous studies, an association between marital status and grip strength has been reported [13, 14], but whether spousal bereavement is causally related to grip strength is as far as we know unknown. Moreover, the causal impact of spousal bereavement on cognition, one of the other pillars of intrinsic capacity, is inconsistent [15]. Some cross-sectional and longitudinal studies report that spousal bereavement is associated with lower levels of cognitive functioning [16–19], while others find no group differences [20–22]. A recent systematic review concludes that spousal loss may be a risk factor for cognitive decline, but that effects may be sex-specific and depend upon time since bereavement [15]. The effect of spousal bereavement on cognitive functioning tends to be attenuated, or even disappears, when taking sociodemographic and health-related factors into account [20].

On average, intrinsic capacity is lower in older age groups, in women and among those with lower socioeconomic position [23]. Lower socioeconomic position and lifestyles related to poor heath are also risk factors for early spousal death [24–26]. Associations of spousal bereavement with cognitive and vitality outcomes are thus likely to be shaped by sociodemographic factors such as age, sex, and socioeconomic position, as well as health behavior and underlying diseases.

In the current observational study, the aim was to examine the effect of spousal bereavement on physical and cognitive capabilities in older age. As there are many potential confounding mechanisms, we make use of a prospective study design and propensity score matching to strengthen conclusions on causality. The current study contributes to the literature by using a large population sample with objective capability measures, longitudinal data, a broad set of health-related adjustment variables, and links to national population registries allowing objective observations of socioeconomic position and spousal death.

## Method

### Study sample

The study sample included participants in the Tromsø study, which is an ongoing population-based health study in Tromsø, Norway, initiated in 1974 [27]. Tromsø is the largest municipality in northern Norway (2015: 73,000 inhabitants) and covers both urban (80%) and rural living areas. In the Tromsø study, both complete birth cohorts and random samples of the population in the area have been invited to seven repeated survey waves so far [28]. The data collection includes questionnaires, interviews, biological samples, and clinical examinations. From Tromsø4 in 1994 and onwards, capabilities such as grip strength and cognition are measured in subsamples of participants completing the main clinical examination in the specific wave. For the present analyses, data from waves 4–7, collected in 1994–5, 2001, 2007–8, and 2015–16, were used.

**Figure 1:**
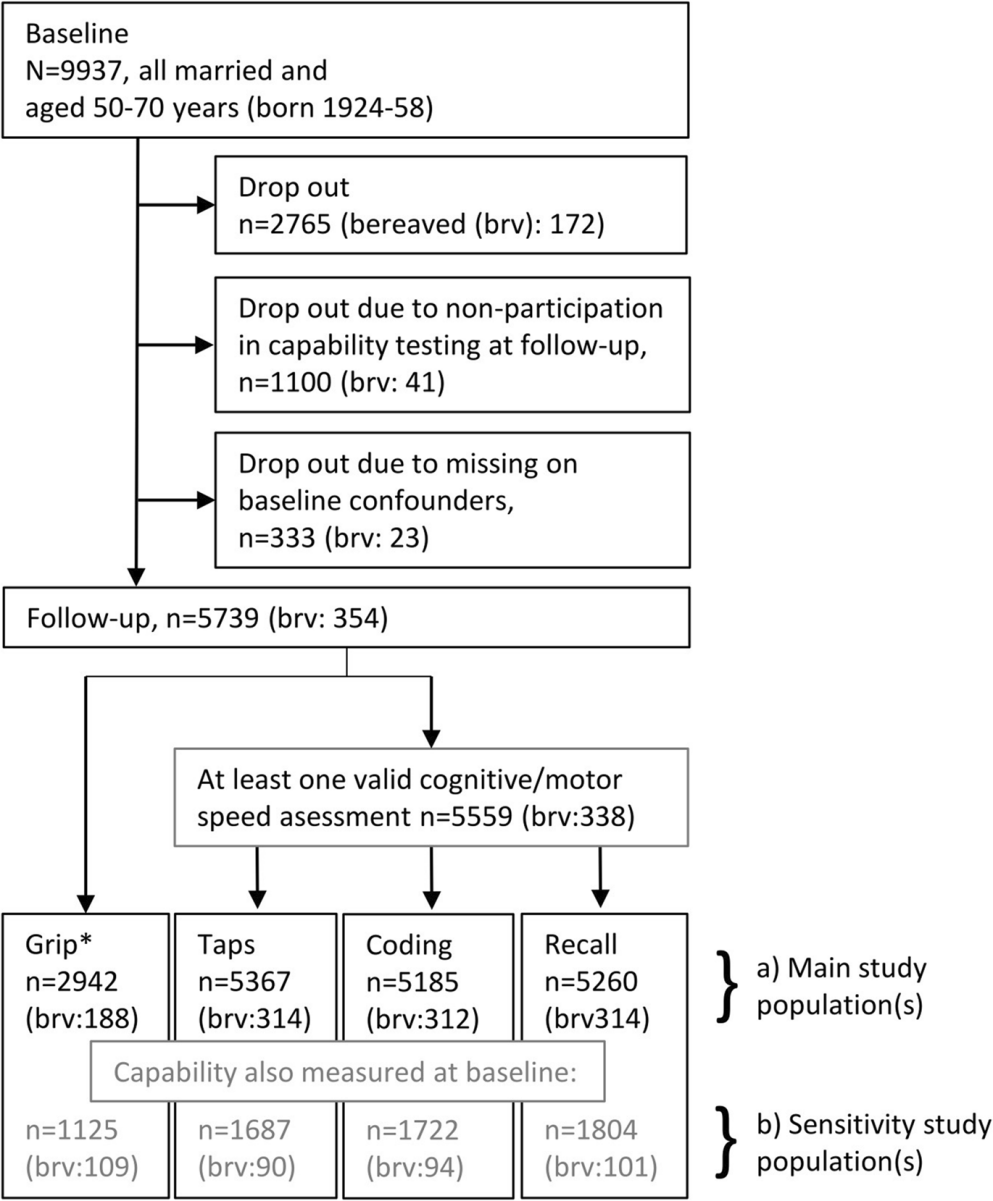
Flow-chart study design. *Grip strength at Tromsø5 was limited to those at age 70+. Thus, a smaller number performed grip strength testing. brv, number of participants experiencing bereavement during follow-up

The study sample encompassed only Tromsø study participants with data from at least two consecutive study waves: Tromsø4–5 or Tromsø6–7 [27, 28]. To boost statistical power, the two samples were combined with baseline at either Tromsø4 or Tromsø6 and follow-up at either Tromsø5 or Tromsø7, respectively. Participants in both samples were between 50 and 70 years at baseline with the first sample being born during 1924–1945 and the latter during 1937–1958. In total, 9937 married participants participated in the baseline surveys at ages 50–70 years. Among these, 5739 participants with valid baseline covariates and measured capability at follow-up were included (see Figure 1, under “Main study population(s)”). Additional sensitivity analyses were performed for participants with capability measurements at both baseline and follow-up, which were one-third of the sample (see Figure 1, under “Sensitivity study population(s)”).

### Study design

**Figure 2:**
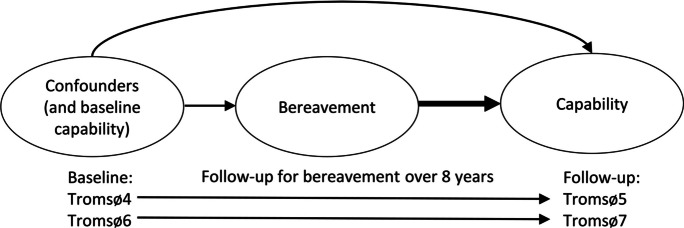
Causal diagram

A historic cohort study design was applied (see the model illustrated in a directed acyclic graph in Figure 2). First, the main exposure, bereavement, was constructed the following way: married/registered partners at Tromsø4 were followed for spousal bereavement for 7–8 years until Tromsø5. Similarly, married/registered partners at Tromsø6 were followed for spousal bereavement for 7–8 years until Tromsø7. For those surviving and participating in the follow-up Tromsø study wave, grip strength and cognitive abilities were assessed. A range of confounders were also assessed at baseline, and in a subset, cognitive and physical abilities were assessed. In this way, by applying a quasi-experimental statistical method called propensity score matching, we were able to study whether spousal bereavement affected subsequent physical and cognitive capability, conditioning on baseline health-related factors and education, as well as on baseline physical and cognitive capability in a subsample (Figure 2).

### Spousal bereavement

Our main exposure variable, spousal bereavement, was based on annual marital status data during 1994–2016 from Statistics Norway and linked to the participants in the Tromsø Study using the unique personal identification number of all Norwegian residents. The classification of marital status used was from 1993 and stratified into the codes 1. non-married, 2. married, 3. widow/widower, 4. divorced, 5. separated, 6. registered partner, 7. separated partner, 8. divorced partner, and 9. remaining partner. In 2009, the Marriage Act was changed to allow same-sex marriages, and the possibility to enter registered partnership was cancelled. Those who already were registered as partners could continue as registered partners, or have their partnership converted into marriage, and hence, all marital status codes 1–9 are still valid. Based on these time series, we could identify year of spousal bereavement and create an indicator with 1 indicating spousal bereavement and 0 indicating no spousal bereavement during the 7–8 years follow-up.

### Physical and cognitive capability

The outcome variables on physical capability were operationalized as grip strength and motor speed from finger tapping. The physical and cognitive capability assessments were performed in the exact same manner at the baseline and follow-up study waves. In Tromsø7, a subsample had grip strength was measured with two devices, and these measures were used to bridge the Jamar measurements (in kilos) to Martin Vigorimeter (bar) using the sex-specific regression equations with the maximum Jamar values of six trials (kg), age at measurement (years), and height at measurement (cm). The equations were as follows: Estimated Martin Vigorimeter value in bar for men = (5.41+Jamar in kg×Age−0.22×Height)/27.49; Estimated Martin Vigorimeter value in bar for women = (14.03+Jamar×Age−0.18×Height)/20.02. The outcome variable on motor speed was assessed with a finger-tapping test in Tromsø5–7. Participants were instructed to tap as fast as possible with the index finger on a keyboard key for 10 s, three times with each hand. The mean number of taps of all six measurements was used. If measurements were missing for one hand, the mean of the other hand was used. To assess performance variability, the standard deviation of the three measurements for the dominant hand was used. These tests are thoroughly validated and widely used for assessment of motor speed [29].

The outcome variables on cognitive capability were operationalized as higher order processing speed and executive functioning, which were assessed in Tromsø5–7 with the digit symbol test, a subtest from the Wechsler Adult Intelligence Scale (WAIS-R) [30]. Using pen and paper, the participants were asked to fill in as many correct symbols of a maximum of 96 as accurately as possible in 90 s [31]. The total number of correctly completed digit symbol pairings was used in this study. The digit symbol coding task is considered a highly sensitive task influenced by aging as well as neurological and mental health issues [32]. Memory was assessed in Tromsø5–7 with immediate recall of the twelve-word test [33]. The same test battery was used at each time point and administered by trained personnel.

Baseline confounders were registry-based data on sex, age (continuous, range 50–70 years), time between study waves (7 or 8 years), and education (primary, secondary, tertiary), as well as information from the Tromsø study: self-reported current state of health (poor, neither good nor poor, good, excellent), current smoking (yes, no), a history of cardiovascular diseases (a dichotomous variable was created with category “yes” if the respondent had any of the diseases heart attack, angina, or stroke, and “no” if respondents had none), and diabetes (yes, no). Mental distress was assessed using the 7-item CONOR-Mental Health Index (CONOR-MHI) at Tromsø4 and the 10-item Hopkins Symptom Check List (HSCL-10) at Tromsø6. The items at both scales ranged from 1 to 4, where 1 indicates no distress and 4 indicates very much or extremely much distress. Mean scores above 2.15 for CONOR-MHI and above 1.85 for HSCL-10 were used to define distress and values below these cut-points were used to define no distress, as suggested in a validation study which included both scales [34]. Missing items were imputed with the mean value on that item. Persons with six or more missing items on the HSCL-10, and four or more on the CONOR-MHI, were set as missing for these tests. Systolic blood pressure (mmHg) was measured using Dinamap Vital Signs Monitor 1846 (Critikon Inc., Tampa, FL, USA) which is a non-invasive, microprocessor-controlled device, and uses the oscillometric method [35]. The proper cuff size was selected based on the circumference of the upper right arm in the individual participant. After 2-min seated rest with the cuff on, three measurements were recorded with 1-min intervals. The mean of the last two measurements was used in the analyses.

### Statistical analysis

Stata SE 17.0 for Windows 64-bit (StataCorp LLC) was used for all analyses. Propensity score matching (PSM), a quasi-experimental statistical method accounting for baseline confounders, was applied to estimate the causal effect of bereavement on each of the outcome measures [36] (Figure 2). In our setting, spousal bereavement was the “treatment” and the (main) exposure in the study. PSM involves selecting units in the sample, so the exposure is independent of the measured covariates in the matched sample. This method can offer advantages in robustness and performance over other methods like propensity score weighting and outcome regression. Thus, the effect was the difference in capability between the scenario in which an individual experiences bereavement and the counterfactual setting in which this individual did not experience bereavement. The study design fulfills the criteria for propensity score matching as we had a temporal data setup with a wide range of baseline confounders; thereafter, bereavement was assessed during the follow-up period, and finally the main outcomes were measured. The advantage of using the propensity score is that the distribution of observed baseline confounders would be similar for both those experiencing bereavement and those who did not. The method assumes that bereavement is exogenous and that the differences in test performance between the bereaved versus the non-bereaved are solely due to bereavement and not due to underlying differences in health [36]. The procedure was performed separately for each outcome employing the user-written Stata command psmatch2 [37]. The first step of the procedure was to estimate propensity scores using logistic regression with bereavement status as the outcome variable. Baseline predictor variables were registry-based sex, age, and education, and survey-based self-reports of overall health, current smoking, a history of heart attack, stroke, angina, diabetes, and mental distress, as well as measured systolic blood pressure (SBP). The same model was used for each outcome. Thus, the aim was to predict bereavement based on a set of baseline predictor variables thought to be associated with both later bereavement risk and test performances. Default radius matching was applied with caliper width as 0.2 SD of the logit of the propensity score [38]. Secondly, individuals were matched based on the similarity of propensity scores. Finally, the average treatment effects on the treated (ATT) were estimated, providing the differences in outcome measures between those experiencing spousal bereavement versus those not experiencing this. Confidence intervals (95%) for ATTs were estimated using bootstrapping with 200 repetitions. Differences in continuous variables between groups were tested using the *t*-test, while differences between categorical variables were investigated using the Chi-square test. The significance level was set to 5%. Logistic regression was used to investigate the association between baseline confounders and spousal bereavement.

Additionally, we performed a sensitivity analysis in a subsample of participants with capability measured at both baseline and follow-up. In this sensitivity analysis, baseline capability was added to the list of confounders, one by one, for the estimation of the propensity scores. So, for example, in the study of whether bereavement affected grip strength, baseline grip strength was added to the covariate list, and likewise in the study of whether bereavement affected finger-tapping performance, baseline finger-tap performance was added to the covariate list. The number in each subset was around one-third of the larger sample and differed according to the capability measure: 1125/2942 for grip strength and 1689/5367 for finger tapping, 1722/5185 for digit symbol test, and 1804/5260 for immediate recall (Figure 1). Due to the substantially lower sample sizes, we denoted these sensitivity analyses, as healthy selection bias might be an issue in these smaller samples. In an additional set of analyses, data was stratified by sex to investigate bereavement by sex interaction effect. Among the baseline participants, not all participated in testing at the next survey wave due to death, random non-invitation, and non-participation due to other causes, and these individuals were thus not part of our study population. This exclusion could be a source of selection bias. Random non-invitation should not be associated with bereavement, and thereby not a source for bias, while the other two causes of non-participation could. To formally test whether non-participation was associated with bereavement, we did a logistic regression analysis among all married baseline participants aged 50–70 years, with spousal bereavement from the national registry as outcome and non-response at follow-up survey as exposure variable, adjusted by age and sex. In an age- and sex-adjusted analysis, non-response was not associated with spousal bereavement (*p*=0.25). Thus, selection bias due to non-response should not be a large issue in our study. Baseline elevated blood pressure, poor self-reported health, heart diseases, diabetes, low education, higher age, and male sex were all significantly related to non-response (*p*<0.05).

## Results

Among the 5739 study participants with at least one capability measure at follow-up, 6.2% experienced bereavement (Figure 1). The bereavement occurred on average 3.7 years (SD 2.0) before the capability assessment. There were similar percentages of bereavement across the different capability measure samples. In bivariate analyses without any control for confounders, the bereaved had significantly lower scores on all the capability outcome measures than the non-bereaved (all *p*-values <0.001).

### Baseline factors and their association with bereavement

In an age-adjusted logistic regression analysis (*n*=5739), women had higher risk of spousal bereavement than men (OR=4.2, 95% CI 3.2, 5.5), and in a sex-adjusted analysis, higher age was associated with higher bereavement risk (per one-year higher age OR=1.11, 95%CI 1.09, 1.14). Adjusted by age and sex, the following variables were associated with spousal bereavement: higher education level was associated with lower bereavement risk (each step from primary, to secondary to tertiary was associated with OR=0.77 (95% CI 0.66, 0.91), excellent self-reported health was associated with lower bereavement risk compared with poor self-reported health (OR=0.46, 95% CI 0.24, 0.88), current smoking was associated with significantly increased risk (OR=1.35, 95% CI 1.04, 1.75), and so was a history of cardiovascular disease (OR=1.37, 95% CI 0.95, 1.97), albeit not statistically significant. Baseline diabetes (*p*=0.58), mental distress (*p*=0.21), and systolic blood pressure (*p*=0.48) were not associated with bereavement.

In the subsample with baseline and follow-up capability measures, the models adjusting for age and sex, baseline grip strength, baseline immediate recall, and finger-tapping speed or variability were not associated with bereavement, but higher baseline scores on the digit symbol coding test (per one-unit higher score OR=0.98, 95% CI 0.96, 0.99) or better baseline immediate recall (per one-unit higher score OR=0.88, 95% CI 0.78, 0.99) were associated with reduced bereavement risk. After adding the remaining baseline confounders to the regression model, the association was only slightly attenuated for the digit symbol coding test (OR=0.98, 95% CI 0.96, 1.00), and more so for immediate recall (OR=0.90, 95% CI 0.80, 1.02).

### Matching on propensity scores

Before matching on propensity scores, those with spousal loss versus those without spousal loss were unbalanced on confounders (Tables 1 and 2), while after matching, the samples were balanced, and there were no significant differences on the included confounders (the *p*-values for *t*-tests ranged from 0.74 to 0.99) (see Figure S1 in Supplement).

**Table 1:** Overview of the baseline characteristics of study participants between 50 and 70 years of age and included in the main and sensitivity analyses with grip strength at follow-up as the capability outcome measure. The sensitivity study population was conducted in a subset of the main population with grip strength measured at both baseline and follow-up

	Main study population (*N*=2942)				Sensitivity study population (*N*=1125)			
	Not bereaved (*n*=2754)		Bereaved (*n*=188)		Not bereaved (*n*=1016)		Bereaved (*n*=109)	
Women, *n* %	1343	48.8	153	81.4	547	53.8	91	83.5
Men, *n %*	1411	51.2	35	18.6	469	46.2	18	16.5
Birth year, min max	1924	1958	1924	1958	1924	1958	1924	1958
Age at baseline, mean (SD)	61.1	(5.8)	64.3	(4.5)	65.3	(4.7)	66.5	(3.5)
Age at follow-up, mean (SD)	69.0	(5.6)	72.1	(4.4)	72.9	(4.6)	74.1	(3.4)
Capability measures at follow-up:								
Grip strength in bar, mean (SD)	0.9	(0.3)	0.8	(0.2)	0.8	(0.3)	0.7	(0.2)
Capability measures at baseline:								
Grip strength in bar, mean (SD)	NA	NA	NA	NA	0.8	(0.2)	0.7	(0.2)
Baseline confounders:								
SBP, mean (SD)	139.3	(20.8)	143.9	(22.8)	146.2	(21.4)	147.2	(22.5)
Education, *n* %								
Primary	608	22.1	64	34.0	312	30.7	43	39.4
Secondary	1364	49.5	102	54.3	514	50.6	55	50.5
Tertiary	782	28.4	22	11.7	190	18.7	11	10.1
Self-reported health, *n* %								
Bad	227	8.2	26	13.8	166	16.3	22	20.2
Neither good nor bad	824	29.9	60	31.9	386	38.0	38	34.9
Good	1354	49.2	91	48.4	384	37.8	44	40.4
Excellent	349	12.7	11	5.9	80	7.9	5	4.6
Daily smoking, *n* %								
No	2343	85.1	152	80.9	858	84.4	93	85.3
Yes	411	14.9	36	19.1	158	15.6	16	14.7
CVD, *n* %								
No	2525	91.7	169	89.9	896	88.2	97	89.0
Yes	229	8.3	19	10.1	120	11.8	12	11.0
Diabetes, *n* %								
No	2644	96.0	181	96.3	973	95.8	105	96.3
Yes	110	4.0	7	3.7	43	4.2	4	3.7
Mental distress, *n* %								
No	2616	95.0	172	91.5	974	95.9	100	91.7
Yes	138	5.0	16	8.5	42	4.1	9	8.3

**Table 2:** Overview of the baseline characteristics of study participants between 50 and 70 years of age and included in the main and sensitivity analyses with cognitive test performance at follow-up as the capability outcome measure. The sensitivity study population was conducted in a subset of the main population with cognitive testing at both baseline and follow-up. The sensitivity population was a subset of the main population with cognition measured at both baseline and follow-up

	Main study population (*N*=5559)					Sensitivity study population (*N*=1810)			
	Not bereaved (n=5221)		Bereaved (*n*=338)			Not bereaved (*n*=1708)		Bereaved (*n*=102)	
Women, *n* %	2633	50.4	267		79.0	884	51.8	85	83.3
Men, *n* %	2588	49.6	71		21.0	824	48.2	17	16.7
Birth year, min max	1924	1958	1924		1956	1937	1958	1938	1956
Age at baseline, mean (SD)	59.9	(5.5)	62.6		(4.9)	60.4	(5.4)	63.2	(4.2)
Age at follow-up, mean (SD)	67.3	(5.5)	70.0		(4.9)	68.4	(5.4)	71.2	(4.2)
Capability measures at follow-up:									
Coding test, mean (SD)	35.1	(12.5)		31.1	(12.0)	39.7	(10.8)	36.5	(10.6)
Finger taps, mean (SD)	53.2	(8.9)		49.7	(9.5)	54.9	(7.5)	52.3	(7.3)
Finger taps variability, mean (SD)*	1.7	(1.8)		1.7	(2.3)	1.8	(1.8)	1.8	(1.4)
Immediate recall, mean (SD)	6.6	(1.9)		6.2	(1.9)	7.0	(2.0)	6.7	(1.9)
Capability measures at baseline:									
Coding test, mean (SD)	NA	NA		NA	NA	42.9	(11.8)	38.9	(11.0)
Finger taps, mean (SD)	NA	NA		NA	NA	54.7	(8.8)	51.0	(9.6)
Finger taps variability, mean (SD)*	NA	NA		NA	NA	1.9	(2.1)	2.4	(3.7)
Immediate recall, mean (SD)	NA	NA		NA	NA	6.9	(1.7)	6.5	(1.6)
Baseline confounders:									
SBP, mean (SD)	140.5	(20.7)		144.1	(21.4)	137.3	(20.2)	139.2	(21.1)
Education, *n* %									
Primary	1518	29.1		133	39.3	295	17.3	27	26.5
Secondary	2509	48.1		163	48.2	899	52.6	60	58.8
Tertiary	1194	22.9		42	12.4	514	30.1	15	14.7
Self-reported health, *n* %									
Bad	1266	24.2		89	26.3	58	3.4	3	2.9
Neither good nor bad	2090	40.0		139	41.1	470	27.5	25	24.5
Good	1519	29.1		100	29.6	939	55.0	64	62.7
Excellent	346	6.6		10	3.0	241	14.1	10	9.8
Daily smoking, *n* %									
No	4107	78.7		257	76.0	1472	86.2	79	77.5
Yes	1114	21.3		81	24.0	236	13.8	23	22.5
CVD, *n* %									
No	4741	90.8		301	89.1	1581	92.6	96	94.1
Yes	480	9.2		37	10.9	127	7.4	6	5.9
Diabetes, *n* %									
No	5064	97.0		330	97.6	1637	95.8	98	96.1
Yes	157	3.0		8	2.4	71	4.2	4	3.9
Mental distress, *n* %									
No	4958	95.0		313	92.6	1625	95.1	93	91.2
Yes	263	5.0		25	7.4	83	4.9	9	8.8

^*^The variability measure for each individual is the SD of three finger taps on dominant hand

### Propensity score matching analysis

In the propensity score matching analyses, there were no significant effects of bereavement on grip strength, immediate recall, or finger-tapping speed/variability, but bereavement had a significant negative impact on the digit symbol coding test (Table 3). The effect size (ATT) of spousal bereavement on the digit symbol coding test was −1.33 (95% CI −2.57, −0.10, *p*-value=0.03) after matching baseline confounders. This translates into an expected outcome on this test of 31.1 points for the bereaved and 32.4 points if spousal bereavement had not occurred. However, when baseline performance on the digit symbol coding test was added to the list of confounders in the subsample analysis, the effect was no longer significant and the point estimate was almost fully attenuated: ATT=−0.04 (95% CI −1.83, 1.75, *p*=0.96). It is worth noting that baseline digit symbol coding test scores were only available for a subset of 1722 out of a total of 5185 participants. The change in the ATT point estimate might be due to the different sample, but this seems unlikely since the ATT in an analysis using the same subsample and leaving out the baseline digit symbol test scores from the list of baseline matching confounders was −1.29 (95% CI −3.38, 0.80, *p*=0.23), which is similar to the result in the larger main sample. The effect sizes were similar in men and women; thus, there was no sex by bereavement interaction.

**Table 3:** Average treatment effect*** of bereavement (ATT) on the outcome measures physical and cognitive capability

	N (#Bereaved, #Not bereaved)	ATT (95% CI), p-value	Expected outcome	
			Bereaved	Not bereaved
Main sample*				
Grip strength (bar)	2942 (186, 2754)	−0.01 (−0.04, 0.03), 0.71	0.81	0.81
Finger-taps	5367 (314, 5053)	−0.47 (−1.53, 0.60), 0.39	49.7	50.1
Finger-taps variability	5367 (314, 5053)	−0.11 (−0.40, 0.18), 0.47	1.69	1.80
Digit symbol	5185 (312, 4873)	−1.33 (−2.57, −0.10), 0.03	31.1	32.4
Immediate recall	5260 (313, 4946)	−0.13 (−0.34, 0.07), 0.21	6.2	6.4
Sensitivity sample**				
Grip strength (bar)	1125 (109, 1016)	0.01 (−0.04, 0.02), 0.58	0.73	0.74
Finger-taps	1689 (90, 1599)	0.17 (−1.22, 1.55), 0.82	52.1	52.0
Finger-taps variability	1689 (90, 1599)	−0.13 (−0.46, 0.20), 0.43	1.79	1.92
Digit symbol	1722 (94, 1628)	−0.04 (−1.83, 1.75), 0.96	36.9	36.9
Immediate recall	1804 (101, 1703)	0.05 (−0.31, 0.41), 0.78	6.7	6.6

^*^Baseline covariate info on age, sex, education, self-reported health (SRH), systolic blood pressure (SBP), smoking, a history of heart attack, stroke, angina, diabetes, and self-reported mental distress

^**^All baseline T0 covariates as in main sample + baseline capability. Baseline capability was only available for a subsample. ATT results in the sensitivity sample, if baseline capability was not included as a confounder for this sample: grip strength, −0.00 (95% CI −0.04, 0.04); finger-taps, −0.12 (95% CI −1.61, 1.36); finger-taps variability, −0.16 (95% CI -0.48, 0.17); digit symbol, −1.29 (95% CI −3.38, 0.80); immediate recall, −0.10 (95% CI −0.50, 0.30)

^***^The effect measure was the average treatment effects on the bereaved (ATT) with 95% confidence intervals (bootstrapping with 200 repetitions) estimated using propensity score matching with baseline (T0) covariates as matching variables and capability at follow-up (T1)

Bereavement is based on yearly follow-up of marital status between T0 and T1 over 7–8 years

*Nt* the number of bereaved individuals, *Nc* the number of controls (not bereaved)

## Discussion

In this large prospective general population study, applying propensity score matching accounting for baseline confounding, we found that spousal bereavement was not associated with subsequent physical capability or cognition.

To our knowledge, our study is the first of its kind to study whether spousal bereavement is causally related to grip strength, and therefore our null finding is not possible to discuss in relation to findings from other studies. Regarding cognitive function, previous cross-sectional and longitudinal studies report inconsistent findings, either that spousal bereavement is associated with lower levels of cognitive functioning [16–19, 39] or no group differences [20–22]. This inconsistency in results may be due to cultural differences. Widowed individuals may face societal pressure to conform to certain grief norms, increasing their emotional distress and physical health implications. A phenomenon known as the “widowhood effect” suggests that the risk of mortality significantly increases in the surviving spouse, especially during the initial period after the death [40]. Possible reasons for this include stress-related physiological changes, reduced self-care, and isolation. Other factor which might differ between studies, and might impact results include the quality of the marriage [41], type of death of spouse [42], age at bereavement [43], cognitive outcome measures [16, 18, 39], length of follow-up after bereavement [16, 44], level of grief [16, 20, 45], and study design and the choice of confounding variables [16–22]. A wide range of statistical models have been employed, such as latent-change modelling [16], ordinary least squares and causal mediation analysis [44], hierarchical multiple regression [14, 20], growth-curve model [17, 18], and propensity score matching [39]. Using a causal inference technique, including adjustment for baseline cognition, we found no significant effect of bereavement on memory, or digital symbol performance, a highly sensitive test of cognitive issues in older adults [46, 47]. At odds with our null findings for memory, bereavement was associated with a negative effect on memory in the Longitudinal Ageing Study Amsterdam (LASA) [16]. This divergence is likely not due to sample size differences or baseline covariates as the studies were similar in these respects, but differences in outcome measure could impact the findings. LASA used a composite cognitive outcome score based on both immediate and delayed recall, while we used only immediate recall. Furthermore, and maybe more important, the LASA study participants were older than in our study (60−85 years versus 50−70 years at baseline). In line with the LASA results, a US-based study, using a composite cognitive index [17], and a Korean longitudinal study, using the Mini-Mental State Examination [18], both reported widowhood/bereavement to be associated with cognitive decline. However, an Icelandic study, which like us used registry-based data on marital status, did not find any long-term effects of bereavement on cognition [22]. One Australian longitudinal study applied two different methods, PSM (like we did) and difference-in-difference (Diff-in-Diff), and they found a modest negative effect of spousal loss on processing speed and memory only for the Diff-in-Diff method, while the PSM method provided no significant effect, in line with our findings [39].

Past studies generally indicate that spousal loss is associated with worse health and social outcomes. However, there is a clear selection—those who experience spousal loss are a non-random group, who regardless of spousal loss would have different health and social outcomes. Our findings, using first the main sample without adjustments for baseline cognition, and then the subsample with adjustments for baseline cognition, show that pre-existing differences influence the likelihood of bereavement, and consequently not adjusting for these could create false significant results or inflate existing association. This is in line with a study reporting that the observed association between bereavement and cognition might be due to confounding by socioeconomic status and health-related factors [20, 48].

Our study illustrated that many baseline variables associated with later cognition, such as age, sex, education, self-reported health, smoking, and heart disease, also were associated with spousal bereavement risk. Not considering these factors may contribute to inflated associations of spousal bereavement with subsequent cognitive capability. In addition, it is necessary to adjust for baseline of the study outcome. As we can see from our findings, we produced a false significant result for digit symbol coding performance if not adjusting for baseline cognition. Thus, digit symbol coding performance was reduced before spousal bereavement and not due to bereavement.

Many of the sociodemographic and health variables at baseline were associated with increased risk of spousal bereavement in the next 8 years. Being older, a woman, or having a lower educational attainment increased the risk, and those experiencing spousal bereavement had poorer self-reported health and were more likely to be current smokers. Cardiovascular disease, systolic blood pressure, diabetes, and mental distress were not significantly associated with risk of spousal bereavement in sex- and age-adjusted analyses.

Age is biologically linked to a higher risk of death and since women live longer than men and tend to marry men who are on average two to three years older [49], the finding that older women were more likely to be bereaved was expected. Educational attainment contributes to socioeconomic position, and it is well known that lower socioeconomic status increases the risk of smoking and several chronic diseases and reduces overall survival, also in Norway [50]. Smoking is particularly closely linked to socioeconomic status in Norway and an important factor for survival disparities between higher and lower status [51]. Since there is a notable spousal concordance on risk factors for disease [52–54], as well as presence of disease and lifestyle factors such as smoking, it is not surprising that the surviving partner had poorer health at baseline than their peers who did not experience bereavement during the same time period.

Two of the capability measures, most notably digit symbol coding and somewhat less immediate recall, were associated with later spousal bereavement in the regression model also after taking age and sex into account. Performance on these tests is sensitive to the confounders included in the model, such as smoking and cardiovascular disease and diabetes, [33, 55, 56] and in our data, the bereavement risk was slightly higher for those with poorer digit symbol coding results also after adding these baseline confounders to the regression model. This finding reiterates that the group experiencing spousal bereavement during follow-up was different regarding cognition already at baseline from the group not experiencing bereavement, which is in line with other studies reporting health declines, including poorer memory, before spousal death [48].

After applying propensity score matching, the two groups became similar on the characteristics associated with a higher risk of bereavement as well as the other potential confounders, demonstrating the power of this methodology. The subsequent analysis revealed lower digit symbol coding performance in those that experienced bereavement compared to the non-bereaved at follow-up. Since baseline digit symbol coding was found to be a potential confounder for spousal bereavement, we interpret this result as a reflection of the underlying differences in the two groups from baseline rather than an effect of bereavement per se. Indeed, the uncorrected bivariate analyses showed that the bereaved had significantly lower scores on the capability outcomes: grip strength, finger-tapping speed and variability, digit symbol coding, and immediate recall than the non-bereaved at follow-up. After the propensity score matching procedure, only digit symbol coding differed, but further inclusion of baseline cognition removed all effects of bereavement on cognition. Since digit symbol coding is a sensitive test of higher order cognitive functions, it might also be sensitive to underlying group differences not considered by the selected variables or the statistical models.

We did not find an interaction between sex and marital status group, in line with several other studies on bereavement and cognition [16, 22]. As fewer men in the sample experienced bereavement, our study might be underpowered to find such an interaction effect, especially in the smaller subsample with capability measures at baseline and follow-up.

### Limitations and strengths

The main strength of this study is the prospective nature of the collection and registration of objective sociodemographic and capability achieved by combining population study data with national registry data and the large sample. However, even if the sample size was large, analyses focusing on the effect of time since bereavement were still underpowered. Furthermore, any short-term effects might go undetected in our study as we follow participants over several years. Earlier studies have revealed short-term effects of spousal bereavement on a range of health measures, which could also apply to our intrinsic capacity outcomes. Thus, it is important to acknowledge that our findings first and foremost apply to long-term effects. The lack of baseline capability data in the main sample was a notable shortcoming, but the baseline capability data were available for a subsample, and the results without adjusting for baseline capability were similar in this subsample and in the main sample, suggesting that the subsample is representative for the larger main sample. To increase statistical power, two birth cohorts were combined: Tromsø4–5 with follow-up from 1994 to 2001 and Tromsø6–7 with follow-up from 2007 to 2016. This combination of cohorts might come with the cost of increased measurement error if study protocols and testing procedures were not identical across study waves. A strength with the Tromsø study is the implementation of identical protocols across study waves. The outcome capability measures were acquired identically for both cohorts, except for grip strength, where two different devices were used. However, some respondents were measured using both types of devices and a bridge coding developed to harmonize the measurements. Nevertheless, this could introduce measurement error and bias any true effects towards null. Although propensity score matching and traditional regression-based methods have provided similar results in some studies, propensity score matching methods are generally preferred, and have been shown to have greater reduction in confounding and outperform traditional regression-based bias-reducing methods such as ordinary least squares regression [57]. A strength with propensity score matching is that it is more likely to achieve a similar distribution of baseline confounders across exposed and unexposed participants compared with regression analysis, and thereby be more similar to a randomized controlled trial [58]. It has also been argued that propensity score matching to a larger extent separates design and analysis, as the focus is to achieve two groups that are similar on all confounders except for the primary exposure, and this work is performed before the outcome of interest is included [59]. Participants living together as cohabitants, and not in registered partnerships, were not included in the current study. Moreover, for those experiencing spousal bereavement, we did not consider remarriages or those becoming cohabitators, which might buffer any potential negative effects of spousal bereavement, and thereby attenuating any true effects. Finally, we conditioned on participation in both a baseline and a follow-up survey, and even if dropout was not related to bereavement, dropout was related to several baseline factors; our sample was healthier than those dropping out. Matching ensures some control for this bias, but there might still be healthy selection bias, which likely attenuates any true effects towards null.

This study reports on the long-term effects of spousal bereavement in married middle-aged and older adults and does not elucidate the impact of grief on our outcomes. Spousal bereavement is a state of having suffered a loss, whereas grief is a natural response to loss [60]. The current study has revealed that for the case of Tromsø, Norway, in the noted period, factors independently associated with spousal bereavement may explain much of the variation in capability observed. The results in our study suggest that spousal bereavement does not have long-term negative effects on the intrinsic capacity components cognition or physical capability.

## Conclusion

Our careful statistical analysis revealed that spousal bereavement does not have long-term effects on the intrinsic capacity components grip strength or cognition to a substantial degree. Future research and interventions should aim to address the myriad challenges faced by this population, fostering resilience and well-being in the face of spousal loss, using high-quality population longitudinal data.

## Supplementary Information

Below is the link to the electronic supplementary material.

Supplementary file1 (DOCX 306 KB)

## Data Availability

The data that support the findings of this study are available from (http://tromsoundersokelsen.uit.no/tromso/) but restrictions apply to the availability of these data, which were used under license for the current study, and so are not publicly available.
