# Supplementary material for: Spousal bereavement and its effects on later life physical and cognitive capability: the Tromsø study
Source: GeroScience. 2024 Apr 9;46(6):6055–69. doi: 10.1007/s11357-024-01150-y (PMC11493887; doi:10.1007/s11357-024-01150-y)
Supplement: Supplementary file 1 — Supplementary file1 (DOCX 306 KB) [file 11357_2024_1150_MOESM1_ESM.docx]

# Supplement

Figure S1. Estimates for the balancing of the matching variables on the outcomes. The mean standardized bias before (dot) and after matching (x) for each covariate. The graph visualizes the extent whether balancing is achieved on the two matched samples, and a value of 0 indicate perfect matching. Balancing is performed for three samples: A) is the grip strength sample (n=2942), B) is the digit symbol coding sample (N=5185), and C is the sample with digit symbol coding at both baseline and follow-up (N=1722).

1. *Grip strength sample (N=2942).*

*
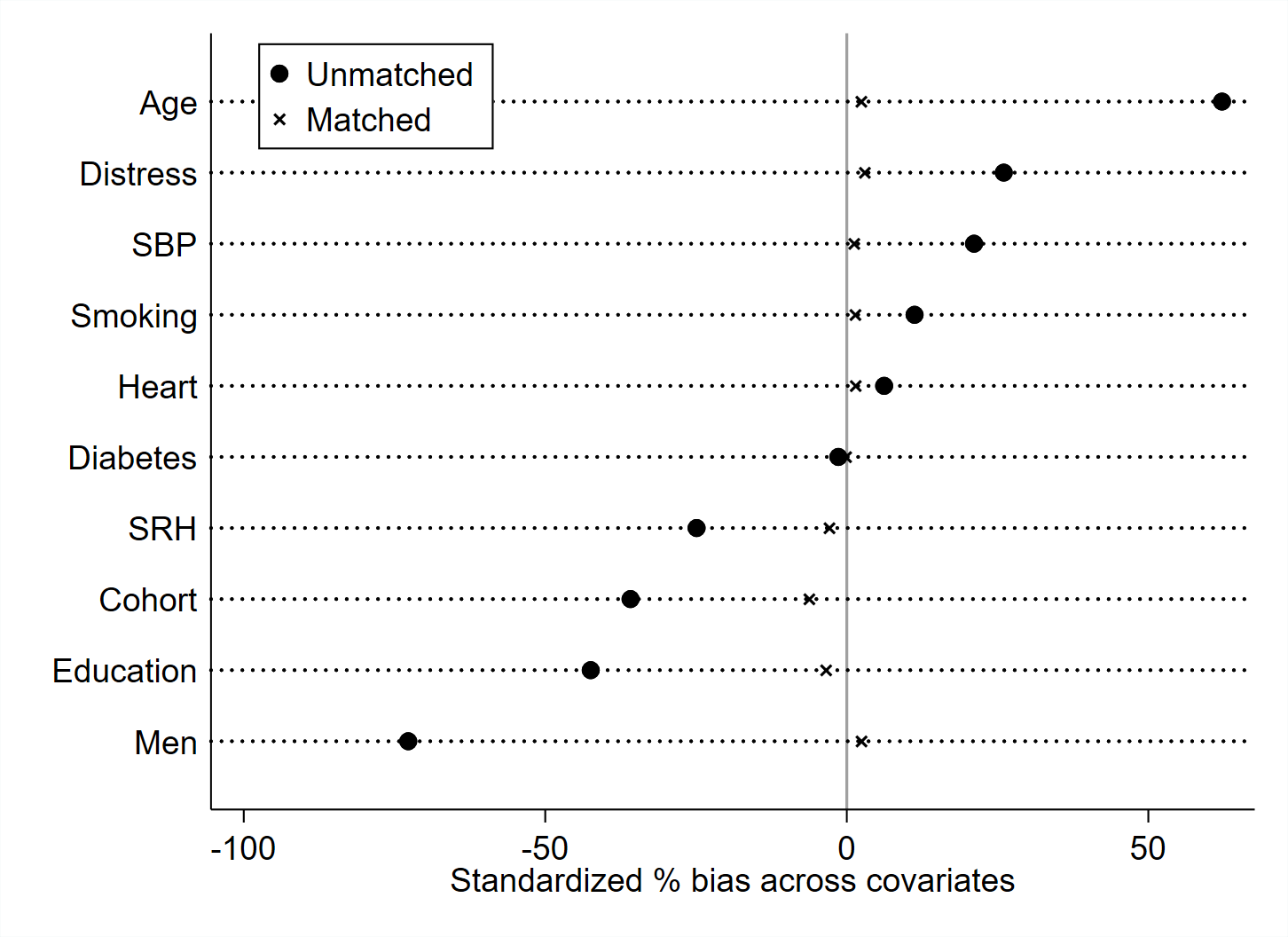
*

For sample A) There were no significant differences between the samples after matching (the p-values for t-tests ranged 0.74-0.99). Before matching there were significant differences for age (p<0.001), mental distress (p<0.001), systolic blood pressure (SBP) (p=0.004), self-reported health (p<0.001), cohort (p<0.001), education (p=0.001) and sex (p<0.001).

1. *Digit symbol coding sample (N=5185).*


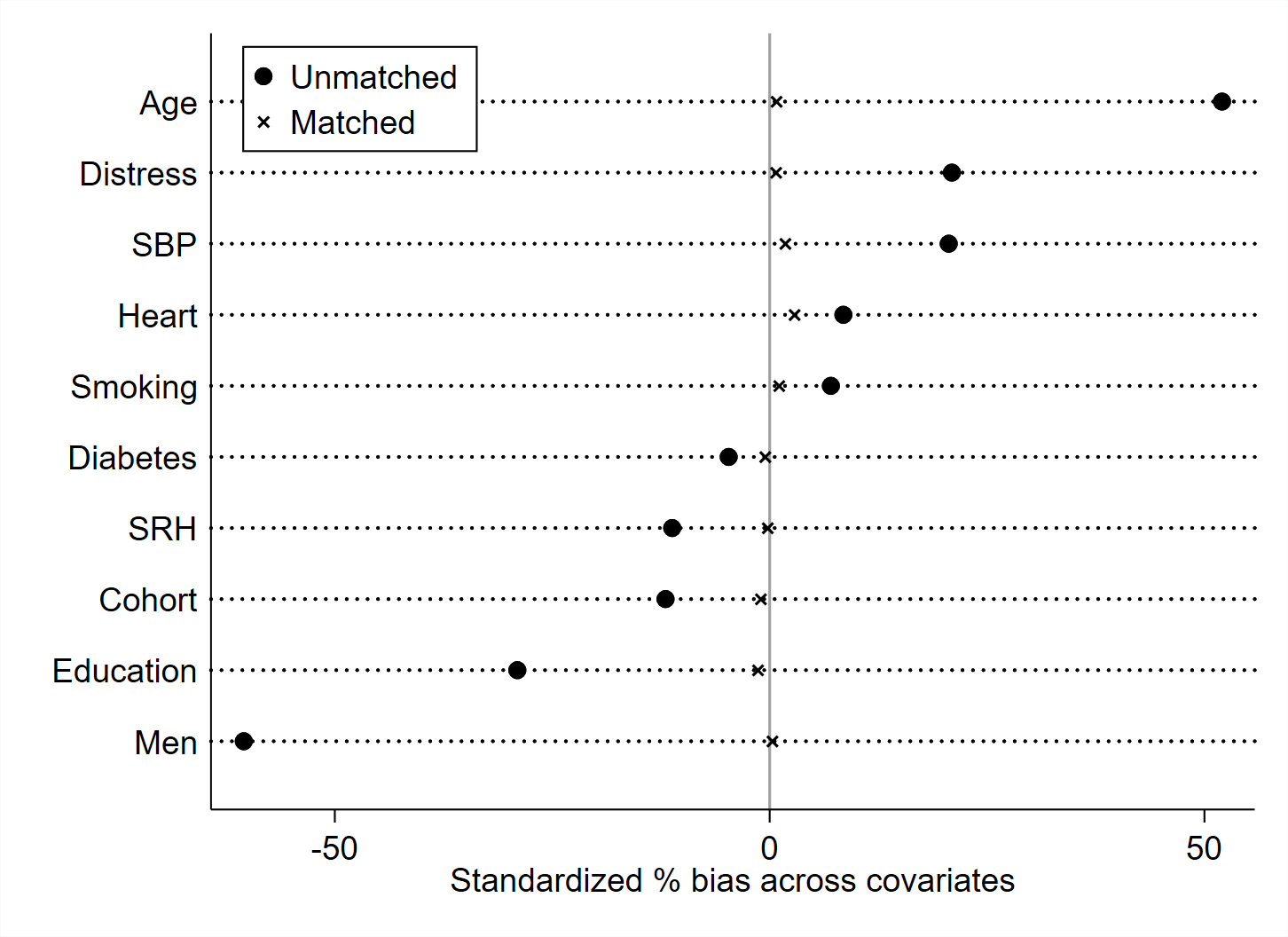


For sample B) There were no significant differences between the samples after matching (the p-values for t-tests ranged 0.73-0.98). Before matching there were significant differences for age (p<0.001), mental distress (p<0.001), systolic blood pressure (SBP) (p<0.001), cohort (p=0.041), education (p<0.001) and sex (p<0.001).

1. *Digit symbol coding sample with baseline digit symbol coding results included (N=1722).*

*
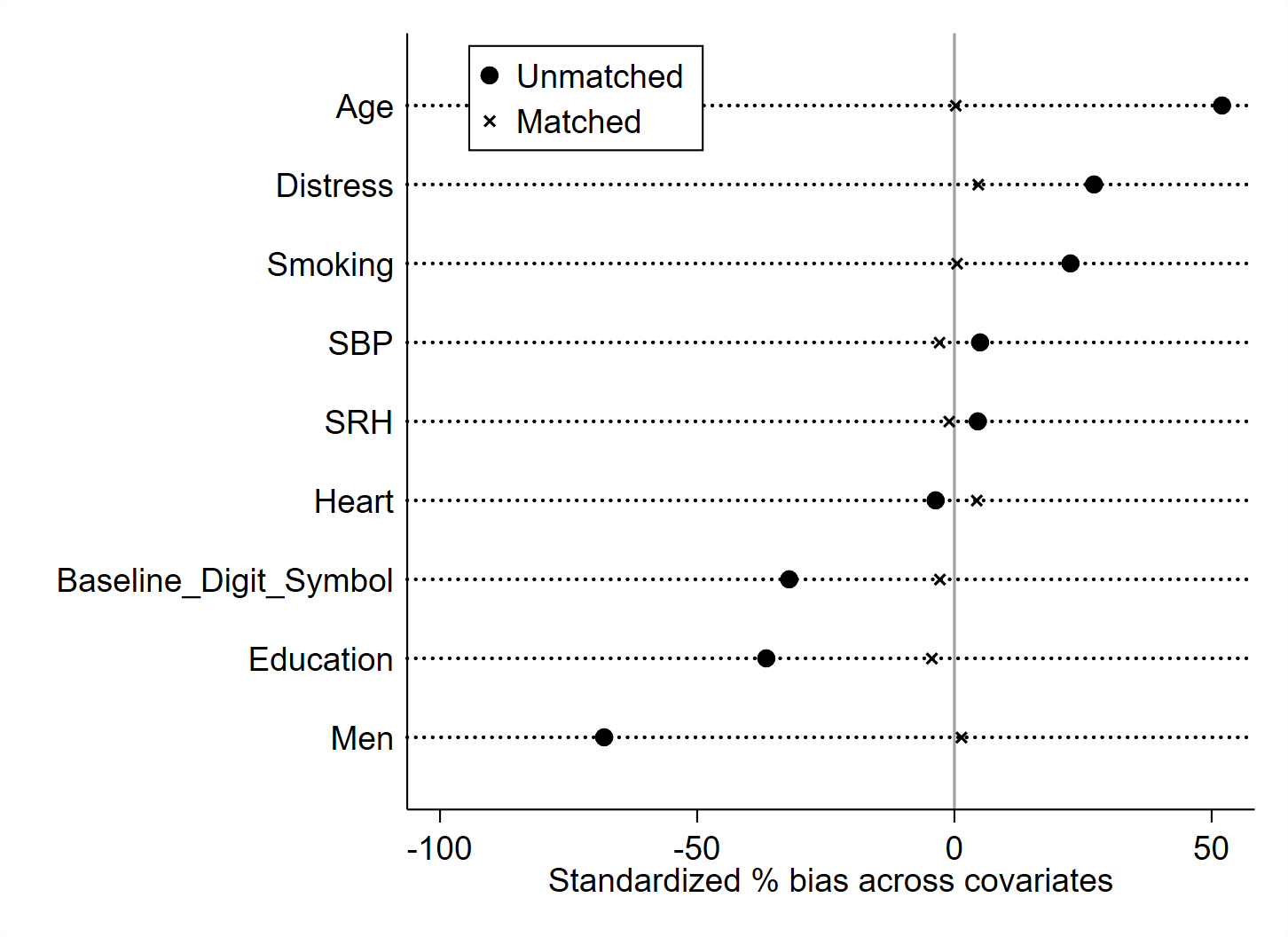
*

For sample C) The % reduction in the mean standardized bias for was 91.1% for baseline coding test performance, and there were no significant differences between the samples after matching (the p-values for t-tests ranged 0.76-0.99). Before matching there were significant differences for age (p<0.001), sex (p<0.001), education (p=0.001), distress (p=0.009), and smoking (p=0.02).
